# Rice Starch Particle Interactions at Air/Aqueous Interfaces—Effect of Particle Hydrophobicity and Solution Ionic Strength

**DOI:** 10.3389/fchem.2018.00139

**Published:** 2018-05-15

**Authors:** Cathy E. McNamee, Yu Sato, Berthold Wiege, Ippei Furikado, Ali Marefati, Tommy Nylander, Michael Kappl, Marilyn Rayner

**Affiliations:** ^1^Faculty of Textile Science and Technology, Shinshu University, Ueda, Japan; ^2^Max Rubner-Institut, Detmold, Germany; ^3^Physical Chemistry, Lund University, Lund, Sweden; ^4^Department of Food Technology, Engineering and Nutrition, Lund University, Lund, Sweden; ^5^Max Planck Institute for Polymer Research, Mainz, Germany

**Keywords:** octenyl succinic anhydride (OSA), starch granules, NaCl, Lagmuir films, optical microscopy, surface forces

## Abstract

Starch particles modified by esterification with dicarboxylic acids to give octenyl succinic anhydride (OSA) starch is an approved food additive that can be used to stabilize oil in water emulsions used in foods and drinks. However, the effects of the OSA modification of the starch particle on the interfacial interactions are not fully understood. Here, we directly measured the packing of films of rice starch granules, i.e., the natural particle found inside the plant, at air/aqueous interfaces, and the interaction forces in that system as a function of the particle hydrophobicity and ionic strength, in order to gain insight on how starch particles can stabilize emulsions. This was achieved by using a combined Langmuir trough and optical microscope system, and the Monolayer Interaction Particle Apparatus. Native rice starch particles were seen to form large aggregates at air/water interfaces, causing films with large voids to be formed at the interface. The OSA modification of the rice starches particles decreased this aggregation. Increasing the degree of modification improved the particle packing within the film of particles at the air/water interface, due to the introduction of inter-particle electrostatic interactions within the film. The introduction of salt to the water phase caused the particles to aggregate and form holes within the film, due to the screening of the charged groups on the starch particles by the salt. The presence of these holes in the film decreased the stiffness of the films. The effect of the OSA modification was concluded to decrease the aggregation of the particles at an air/water interface. The presence of salts, however, caused the particles to aggregate, thereby reducing the strength of the interfacial film.

## Introduction

Oil in water (O/W) emulsions can be stabilized by adsorbing particles at the oil/water interfaces, i.e., the so-called Pickering type emulsions (Pickering, 1907). Particles can also stabilize foams without the use of surfactants by adsorbing to the air/liquid interfaces of the gas bubbles that are suspended in a liquid continuous phase (Chevalier and Bolzinger, 2013). Other systems where an air/liquid interface is stabilized by particles are liquid marbles (Aussillous and Quéré, 2001; Bormashenko, 2011) and colloidosomes (Dinsmore et al., 2002; Thompson et al., 2015). In particle stabilized systems, the interactions between particles at and with the hydrophobic (e.g., oil or air)/aqueous interfaces are crucial. The attachment of particles to these interfaces has been shown to be affected by the surface chemistry, charge, size, and shape of the particle (Aveyard et al., 2000; Kralchevsky and Nagayama, 2000; Schultz et al., 2006; Min et al., 2008; Bleibel et al., 2013). The surface chemistry controls the stability of the particles at the interface and their tendency to aggregate at the interface. A particle that is too hydrophilic will readily detach from the interface into the aqueous phase, causing the emulsion to become instable. A particle that is too hydrophobic will cause the particles to aggregate together and clump at the interface, causing defects such as holes in the film at the interface. Areas of the bare oil/aqueous interface generally result in a less stable emulsion. The surface groups on the particles and their degree of dissociation will contribute to the charge on the particles. An increased charge increases the inter-surface electrostatic repulsions at the interface, which acts to reduce the aggregating and clumping of the particles at the interface. Increasing the particle size increases the magnitude of the inter-surface van der Waals attractive force and the inter-surface attractive capillary force. These forces may cause the particles to aggregate and clump, causing holes in the film of particles at the interface.

Starch is a carbohydrate produced by most green plants to store energy. It consists of a large number of glucose units joined by glycosidic bonds. The resulting polysaccharides (linear amylose and branched amylopectin) are efficiently packed in the plant cells into semi-crystalline starch granules. Starch granules (referred to as “starch particles” from here on) can be isolated from a variety of plants, mainly from tubers and cereals. They have a considerable natural variation with respect to granule size (0.5 to 100 μm), shape (round, oblong, sharp edged polyhedral, etc.) and composition (the ratio between the two constituent polymers of the starches, i.e., amylopectin and amylose) depending on its botanical origin (Jane et al., 1994). Starch particles have been used to stabilize O/W food emulsions (Li et al., 2013; Song et al., 2014; Marefati et al., 2017). This is because starch is a naturally occurring polysaccharide that is safe to use in foods (Sweedman et al., 2013) and because it is abundant, biodegradable, and inexpensive (Hui et al., 2009). Native (non-modified) starch, however, has limitations in its applications, due to its hydrophilic surface properties, which makes it less suitable as a stabilizer. Chemical or physical modifications of the starch particles to change the physical and chemical properties of the starch are common, as this improves the applicability of starch in the food industry as well as for other applications like those used in pharmaceutical technologies.

**Figure 1:**
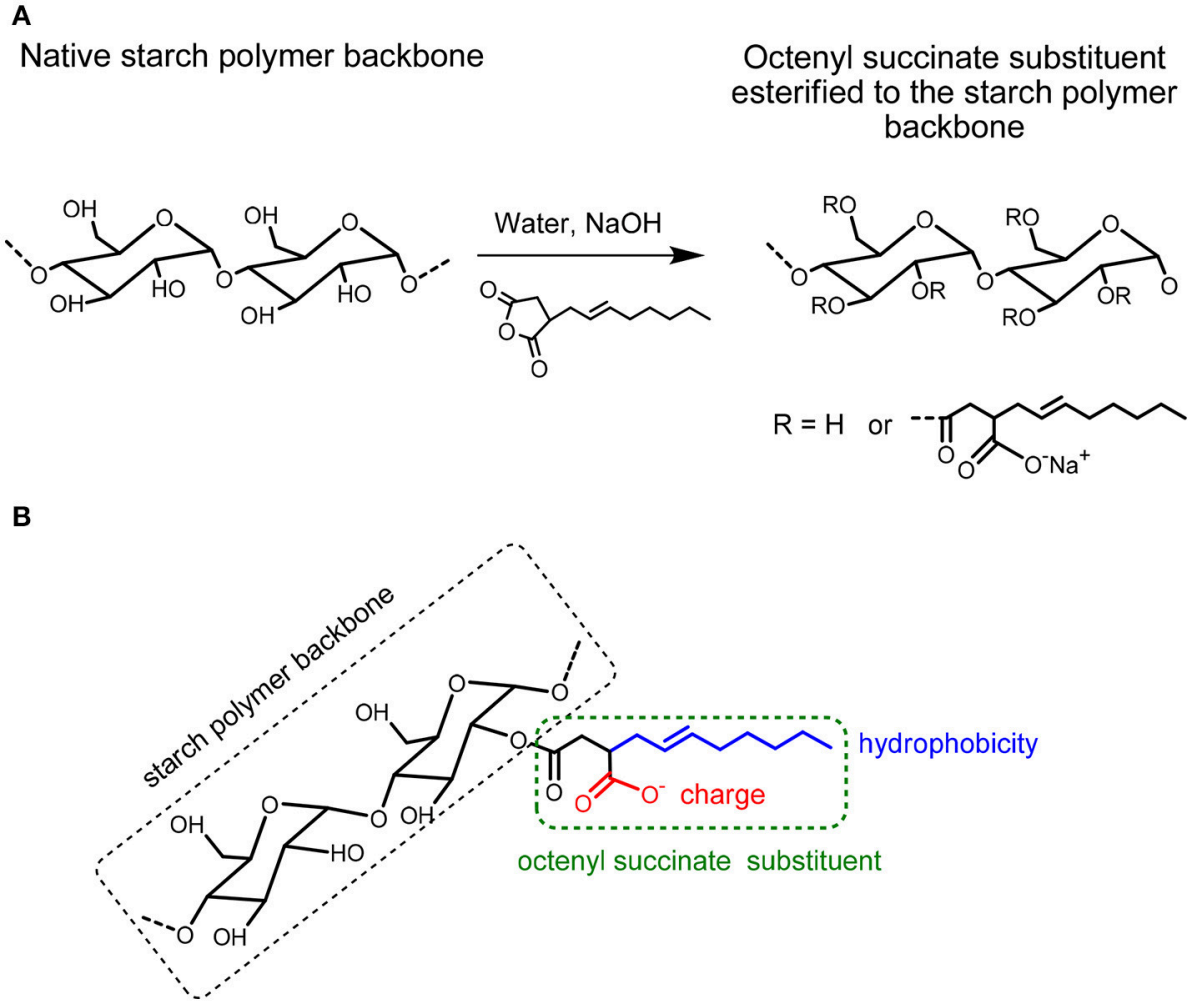
**(A)** Modification of starch by Octenylsuccinic Anhydride (OSA) modification. **(B)** Hydrophobicity (blue color) and possible charge (red color) in the octenyl succinate substituent esterified to the starch polymer backbone.

The emulsifying capacities of native starch particles can be improved by increasing the hydrophobicity of the starch particles via chemically modifying the starch. Octenyl succinic anhydride (OSA) modified starch (OSA starch) is obtained from the esterification reaction between the hydroxyl (OH) groups on starch and octenylsuccinic anhydride, see Figure 1A (Sweedman et al., 2013). An increase in the number of OH groups substituted by OSA has been reported to increase the hydrophobicity of the otherwise hydrophilic native starch (Miao et al., 2014). As the hydrophilicity of the starch backbone is retained (Ovando-Martinez et al., 2017), this reaction can create amphiphilic particles with unique properties. The most widely described synthesis pathway is a reaction in aqueous medium under mild alkaline conditions with the starch in its granular form (Trubiano, 1986). The level of OSA modification is commonly reported by the percentage of OSA used based on the dry weight of starch or the degree of substitution (DS), which is the average number of octenyl succinate (OS) derivatives per glucose unit. The substitution with OSA can occur at carbon 2, 3, and 6 in the glucose molecule of starch (Figure 1A) (Nilsson and Bergenståhl, 2007b; Sweedman et al., 2013). The OH-group at carbon 6 is esterified preferably because of the steric hindrance of the other two OH-groups. A degree of substitution (i.e., the average number of esterified hydroxyl groups per monosaccharide unit) of ~0.02 is typical for commercial OSA starches (Shogren et al., 2000). OSA groups are thought to be mostly present in the amorphous parts of the amylopectin molecule in the interior of the starch. However, they may also exist on the exterior of the granule (Shogren et al., 2000). Starches modified with octenyl succinic anhydride (OSA) have been used in a range of formulations, particularly as a food additive, over the past 50 years. OSA modified starch (E1450) is approved for food applications with a degree of modification up to 3% based on the dry weight of starch (Tesch et al., 2002; Rayner et al., 2012b). For this reason, OSA is generally studied at intervals less than 3%. Rice, corn, tapioca, potato, amaranth, and wheat starch particles have been successfully modified using OSA (Saari et al., 2016; Whitney et al., 2016; Marefati et al., 2017; Ovando-Martinez et al., 2017). There are also reports that show such OSA modified starch particles can be used to stabilize O/W emulsions (Saari et al., 2016; Marefati et al., 2017).

The physical properties of OSA modified starch particles affect their ability to effectively stabilize O/W emulsions. The effect of the OSA modification on the physical properties of starch particles has been studied. The hydrophobicity of a starch particle is reported to increase after the OSA modification. This modification not only increases the amphiphilicity of the starch particle, but also weakens the internal hydrogen bonding of the starch particle (Ovando-Martinez et al., 2017). These effects cause the physical properties of the starch particles to change. For example, the OSA modification of a starch particle has been reported to disrupt the crystalline structure of the starches, causing the OSA starches to have a lower gelatinization temperature than their native starches (Sweedman et al., 2013). In general, OSA starch gels have also been found to be softer than the native starch gels (Ovando-Martinez et al., 2017). The OSA modified rice starch particles have also been shown to aggregate less than the native rice starch (Ovando-Martinez et al., 2017).

The properties of starch particles affecting their behavior at and with a hydrophobic (oil)/hydrophilic (aqueous) interface and the effect of the OSA modification of starch particles is less understood. Studies have shown that the degree of modification of the starch particle, the weight percent of particles used in the system, and the size of the starch particles can affect the surface coverage of the oil/water interface by the particles and the stability of the droplet (Yusoff and Murray, 2011; Li et al., 2013; Marefati et al., 2017). The stability of the droplet with adsorbed particles generally increases as the coverage of the droplet by the particles increases. This is due to the electrostatic interactions that act between two particle stabilized droplets, whose adsorbed particles are charged, and also due to the steric repulsions resulting from the finite size of the particles at the oil/water interface. The OSA modification has been reported to change the charge of starch (Nilsson and Bergenståhl, 2007a; Miao et al., 2014). Thus, the interactions between the particles at the hydrophobic/hydrophilic interface is expected to change with the OSA modification of the starch particles. Additionally, the ionic strength of the aqueous phase is also expected to affect the magnitude of the attractions between charged starch particles in the interfacial layer. This is because the electrostatic repulsions between two charged surfaces in aqueous solutions are screened by salts (Israelachvili, 2011), causing the inter-surface force to be more attractive in solutions of high ionic strength. The addition of salt can therefore potentially be used to increase the particle packing density at the oil/water interface and to improve the packing of the particles at the interface.

In spite of the numerous studies into the physical properties of starch particles modified by OSA and their interactions at and with hydrophobic/hydrophilic interfaces, the effects of the OSA modification of the starch particles and the addition of salt to the aqueous phase on these interactions are still not well-understood. For this purpose, we have systematically studied here the effect of OSA modification of starch particles on the colloidal interactions in films of starch particles deposited at hydrophobic/aqueous interfaces in the absence and presence of salt. This was achieved by modeling the hydrophobic/aqueous interface with an air/aqueous interface. The advantage of using an air/aqueous interface is that the effect of the particle type, particle packing density, and the presence of salts in the aqueous phase on the physical properties (inter-particle interactions, particle aggregations, interfacial stiffness, etc.) of the air/aqueous interface can be systematically studied. Here, we used a combined Langmuir trough and optical microscope system, and the Monolayer Interaction Particle Apparatus (MPIA) (McNamee et al., 2015; McNamee and Kappl, 2016; Azakami et al., 2017), which allows the interactions between the particles to be studied at the same time as the particle packing density and bulk phase properties are varied. In this study, we compared native rice starch particles (NRS) and rice starch particles that were modified with OSA to different degrees. This starch type was chosen, as the size of the rice starch particles (diameter = 6.92 ± 0.17 μm) was large enough to allow their visualization at the air/aqueous interface with an optical microscope. As many food applications of the starch particles also include salt, the effect of salt on the physical properties of the air/aqueous interface in the presence of OSA modified starch particles was also determined. This also allowed us to reveal the impact of electrostatic forces on the particle interactions. The results of this study will help reveal the effect of OSA modification of starch particles on the physical properties of hydrophobic/hydrophilic interfaces in the presence of starch particles. This information is envisioned to improve the use of the starch particles in food applications.

## Experimental

### Materials

The materials in this experiment were chloroform (CHCl_3_, 99.0% purity, Wako Pure Chemical Industries, Japan), sodium chloride (NaCl, JIS Special Grade, Wako Pure Chemical Industries, Japan), and ethanol (EtOH, JIS Special Grade, Wako Pure Chemical Industries, Japan). The water used in this study was ultrapure water (Direct-Q 3 UV, Millipore, USA), which had a specific resistivity of 18.2 MΩ cm.

#### Preparation of OSA modified rice starch and determination of the degree of modification

Rice starch was isolated in a semi-technical scale. Briefly, 8 kg of rice were steeped in 16 kg of a 0.4% NaOH-solution for 16 h at 4°C, in order to soften the endosperm and to enhance protein solubilization. The supernatant was then separated, 30 kg of fresh water added and the rice wet milled with a colloid-mill (150 μm). Afterwards, the protein and the fiber were separated from the starch by repeated centrifugation (decanter) and wet-sieving (vibration sieve) steps. Finally, the starch suspension was neutralized and spray dried using a spray dryer (type minor production, Niro A/S, Kopenhagen, Denmark) (Marefati et al., 2017). Next, 50.0 g of the starch was suspended in 200.0 g of distilled water. The pH-value was adjusted to 8.2–8.4 and maintained constant during the reaction by addition of a 0.5 N NaOH solution. A solution of OSA in acetone (100 mg OSA/mL solution) was added at 32°C with intensive stirring for 5–40 min. The mixture was still stirred until the reaction had finished (90–120 min). The pH did not decrease further with time after the reaction had finished. The amount of OSA in relation to starch dry matter (44.7 g) was 0.6, 1.2, 1.8, 2.4, and 3.0% by mass (Marefati et al., 2017). The modified starch was isolated by centrifugation (7 min, 3,350–5,580 × g), washed with 350 mL of distilled water (5 min suspension followed by centrifugation) and finally with 300 mL of acetone. The third sediment was first dried at room temperature overnight and then in a convection dryer at 30°C for 4 h. Finally, the products were conditioned to their equilibrium moisture content for 2 days at room temperature. All yields varied between 50.1 and 51.1 g (Marefati et al., 2017). The degree of substitution (DS) of these starches was determined by hydrolysis of 2 g of the modified starch with 60 mL of distilled water and 20.00 ± 0.03 mL of a 0.1 N NaOH at 35°C for 24 h in a closed Erlenmeyer flask and by back titration of the excess sodium hydroxide with 0.1 N H_2_SO_4_ to pH = 7.0 ± 0.1. The starch/water-suspension was adjusted to pH = 7.0 ± 0.1 before the addition of the sodium hydroxide solution (Marefati et al., 2017).

The native rice starch particles (NRS) and rice starch particles modified by OSA to different degrees were used in this study. The rice starch particles were modified with OSA to give surface hydrophobized starches with a modification level of 0.46 ± 0.01, 0.97 ± 0.03, 1.40 ± 0.05, 1.90 ± 0.05, 2.36 ± 0.02% OSA by mass in dry matter of the sample. These values correspond to degrees of substitution (DS) of 0.0036, 0.0077, 0.0108, 0.0149, and 0.0186, respectively (Marefati et al., 2017). In this study, we refer to these particles as 0.6RS, 1.2RS, 1.8RS, 2.4RS, 3.0RS, respectively. The diameter of the native rice particles was determined in earlier studies to be 6.92 ± 0.17 μm (Marefati et al., 2017). Scanning Electron Micorspy images from that studied also showed that the NRS and 3.0RS particles had comparable sizes and shapes (Marefati et al., 2017). As the same batch of rice particles used in this present study was the same as that used in these previous studies, the size of the rice particles was treated as 6.92 ± 0.17 μm.

### Preparation of the NRS and RS starch particle spreading solutions

The starch particles were firstly dispersed in ethanol to give a concentration of 7.97 × 10^4^ ± 0.24 × 10^4^ starch particles per μL of ethanol by adding ~0.05 g of the dry particles to 2.5 mL of the ethanol. This solution was then sonicated for 10 min, so as to disperse the particles in the ethanol. Ethanol acted as the solvent to spread the particles at the air/aqueous interface. Organic solvents, such as chloroform, which are commonly used to spread and prepare insoluble Langmuir films at air/aqueous interfaces could not be used in this study, because the starch particles were seen to aggregate in chloroform. Ethanol, however, allowed the starch particles to be well-dispersed. Ethanol also has a positive spreading coefficient (Barnes and Gentle, 2005), which enabled it to spread at an air/water interface and form the films of the starch particles.

### Methods

#### Langmuir trough

A Langmuir Trough (Large microscopy Langmuir trough, Nima Technology Ltd, Coventry, UK) was used to prepare the films of starch particles at the air/aqueous interface. The surface pressure was measured using a Wilhelmy plate of wet filter paper (Barnes and Gentle, 2005) (No.2 240 mm, Toyo, Japan) that was attached to a strain gauge (Nima PS4 surface pressure sensor, Nima Technology Ltd, Coventry, UK).

The Langmuir trough was cleaned with chloroform and then with ethanol, before the surface pressure-area isotherms were measured. Water was next added to the trough and then removed, in order to remove any solvent remaining in the trough. The solution that was to be used as the subphase was subsequently added to the trough and the barriers compressed to maximum. The liquid surface was then cleaned by suctioning the water surface between the two barriers, after which the barriers were fully expanded. The temperature of the subphase was maintained at 20°C by controlling the temperature of the experimental room. Next, 2,000–2,250 μL of the starch particles in ethanol solution was spread drop-wise onto the water surface by using a 1,000 μL syringe (Hamilton, Switzerland). A time of 10 min was allowed for the ethanol to evaporate. The particle films were then compressed with a speed of 80 cm^2^ min^−1^. The surface pressure-area isotherms were recorded during this time. Each isotherm was measured a minimum of three times, in order to ensure the reproducibility of the results.

The surface pressure (∏)-area isotherms were converted to ∏-area per starch particle (*A*_*starch*_) isotherms so as to enable the isotherms of the different starch particle types to be directly compared. This was necessary, as different volumes of spreading solutions needed to be spread to the air/aqueous interfaces for the different particle types, so as to measure both the loose packing and tight packing regions in each film. The area values were converted to area per starch particle (*A*_*starch*_) values by calculating the number of starch particles spread at the air/aqueous interface (N_starch_). This was achieved by using

(1) $$\begin{array}{l} {\text{N}}_{\text{starch}}=\frac{\rho_{\text{starch}}\;V\;\rho_{\text{EtOH}}}{{\text{W}}_{\text{starch}}} \end{array}$$

The parameters ρ_starch_, V, and ρ_EtOH_ are the weight fraction of the starch particles in the ethanol spreading solution, the volume spread at the air/water interface and the density of ethanol (0.789 g/mL), respectively. W_starch_ is the mass of one particle and was calculated using

(2) $$\begin{array}{l} {\text{W}}_{\text{starch}}\;=\;\frac{\rho_{\text{starch}}4\pi {\text{R}}^{3}}{3} \end{array}$$

Here, ρ_starch_ is the density of starch, approximated by the standard value of 1.5 g/cm^3^ (Odeku and Itiola, 2007), and R is the radius of one particle.

The maximum volume fraction of close-packed spheres is reported to be 0.74 (Israelachvili, 2011). Thus, the area expected for a hexagonal packed layer of particles (A_hex_) with mean starch size was calculated using

(3) $$\begin{array}{l} {\text{A}}_{\text{hex}}=\frac{\pi {\text{R}}^{2}}{0.74} \end{array}$$

#### Optical imaging of films of starch particles at air/aqueous interfaces

The combined optical microscope-Langmuir trough set-up used in this study has been described elsewhere (Ngyugen and McNamee, 2014). The Langmuir films were prepared in the same way described to measure the surface pressure-area isotherms. Briefly, the starch particles in ethanol spreading solutions were applied to the air/aqueous interface by using a 1,000 μL syringe (Hamilton, Switzerland), and 10 min was allowed for the solvent to evaporate. The films were then compressed to the desired surface pressure. The surface pressure was maintained at the desired value by using the surface pressure control option on the Langmuir trough, during which time the images of the air/aqueous interface were taken. The images were taken in the order of low to high surface pressures.

#### Monolayer interaction particle apparatus (MPIA)

The colloid probes to be used in the MPIA measurements were prepared by using a light microscope (BX51, Olympus) and a micro-manipulator (Model MMO-202D, Narishige) to attach a rice starch particle (native or OSA modified) to a gold-plated Si_3_N_4_ cantilever (V-shaped, nominal spring constant *k* = 0.15 N/m, OTR8-PS-W, Olympus) with an epoxy resin (Araldite Rapid).

The MPIA was used to measure the forces between a film of starch particles at an air/aqueous interface and the colloid probe in the water. The MPIA is comprised of a force measurement unit that is attached to a Langmuir trough (Riegler & Kirstein GmbH, Potsdam, Germany). Detailed information about the MPIA can be found elsewhere (Gillies et al., 2005; McNamee et al., 2011).

The MPIA was used in the following way. First, the Langmuir trough was cleaned using chloroform and ethanol. Any remaining solvent was eliminated from the trough by adding water to the trough and then by removing it. The colloid probe was next inserted into the MPIA cantilever holder and the cantilever holder attached to the MPIA. Water was then added to the trough and its surface suctioned cleaned. The temperature of the subphase was maintained at 20.0 ± 0.1°C by running thermostated water through the base of the trough by using a circulation system (C25P, ThermoHaake, Karlsruhe, Germany). A calibration factor (CF_mica_) was required to convert the raw force curves (force [V] vs. piezo position [nm] curves) to the calibrated force curves (force [nN] vs. piezo position [nm] curves). This was achieved by measuring the force curves between the probe and a clean mica substrate that was placed across the edges of the Langmuir trough filled with water. CF_mica_ was obtained from the constant compliance region (the region where the probe was in contact with the mica substrate) of these force curves. The water in the trough was removed after the calibration. The solution to be used as the subphase was then added to the trough, and that surface cleaned. The particle in ethanol spreading solution was next spread at the air/aqueous interface, after which 10 min was allowed for the ethanol to evaporate. The films were then compressed to a certain surface pressure, and the forces measured between that film and the same probe that was used to measure CF_mica_. A minimum of 50 force curves was recorded at each surface pressure. CF_mica_ was used to convert the raw force curves (force [V] vs. piezo position [nm] curves) measured between the film of starch particles at the air/aqueous interface and the probe in the aqueous phase to the calibrated force curves (force [nN] vs. piezo position [nm] curves). Zero force was defined at large probe-film separations, where no surface forces acted on the cantilever. Zero distance was defined at the onset of the linear contact region in the force curves; the contact region was defined as the area where the probe was in contact with the particle film at the air/aqueous interface.

The magnitude of the adhesive force between the probe and the film of starch particles at the air/aqueous interface was obtained from the force curve minimum that was measured in the retraction force curves. The average adhesion force (*F*_*ad*_) was calculated by fitting a Gaussian curve to a histogram of the adhesive forces that were calculated from the measured force curves taken at the same conditions (same surface pressure, same particle type, and same subphase solution conditions). The effective stiffness (*S*_*N*_) (McNamee et al., 2010) of the film of starch particles at the air/aqueous interface was calculated by dividing the slope of the linear contact region of the force curves measured between the probe and film of starch particles at the air/aqueous interface (*S*_1_) by the slope of the linear contact region of the force curves measured between the same probe and the mica substrate in water (*S*_2_).

#### Zeta potential measurements

The Zeta potential (ζ) values of the starch particles were measured with a Zetasizer (Zetasizer Nano ZS, Malvern Instruments Ltd, Wostershire, UK) by preparing a 1,000 ppm concentration of native or modified starches dispersed in water or aqueous solutions containing NaCl. Briefly, the electrophoretic mobility was measured for each sample five times. These values were then converted into zeta potential values by using the Helmholtz-Smoluchowski approximation, assuming that the particles were spherical and large compared to the Debye length of the system. The zeta potential values of each sample type were then averaged.

## Results and discussion

### Effect of the degree of modification of the rice starch particle on the physical properties of a film of the rice particles at an air/water interface

**Figure 2:**
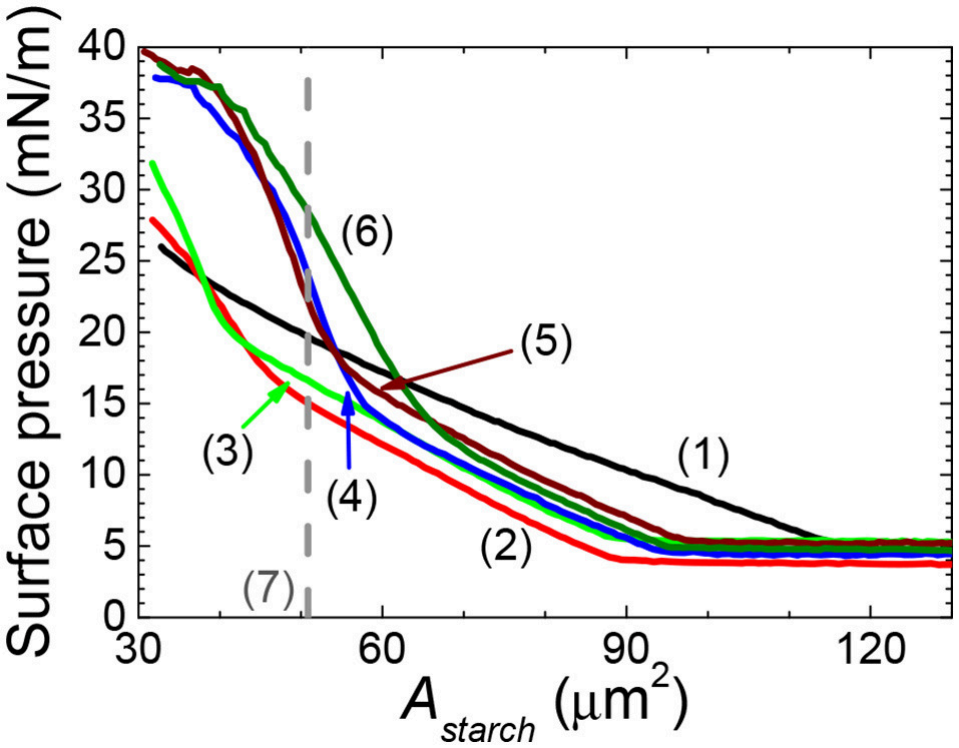
Surface pressure-area per rice starch particle (*A*_*starch*_) isotherm of films of native and OSA modified rice starch particles spread at an air/water interface. NRS: black solid line (1); 0.6RS: red solid line (2); 1.2RS: green solid line (3); 1.8RS: blue solid line (4); 2.4RS: wine color solid line (5); 3.0RS: olive color solid line (6). Area expected for a hexagonal packed layer of particles with mean starch size is shown by the dashed line (7).

The effect of modifying the structure of rice starch on its ability to form films of starch particles at air/water interfaces was firstly investigated by spreading native rice starch particles (NRS) and OSA modified rice starch particles at air/water interfaces and then by measuring their surface pressure-area per rice starch particles (*A*_*starch*_) isotherms, see Figure 2. The rice starch particles that were modified with an OSA modification percentage of 0.46 ± 0.01, 0.97 ± 0.03, 1.40 ± 0.05, 1.90 ± 0.05, 2.36 ± 0.02 are referred to as 0.6RS, 1.2RS, 1.8RS, 2.4RS, 3.0RS, respectively. The NRS particles gave positive surface pressure values for the *A*_*starch*_ values < ~120 μm^2^. The surface pressure increased gradually with an *A*_*starch*_ decrease, suggesting a loose particle packing in the film of the NRS particles at the air/water interface. OSA modification of the rice particles caused the isotherms to shift to lower *A*_*starch*_ values than those measured with the NRS particles, when the same surface pressure values were compared. The isotherms also showed a change in slope from a flatter to a steeper isotherm as the particles were compressed. These two slope regions are thought to indicate films of loose packing and tight packing, respectively. The *A*_*starch*_ values where the phase transition from loose to tight packing films occurred moved to higher values as the degree of modification of the rice particles increased. A change in the inter-particle interactions and therefore the particle packing at the air/water interface may explain this shift in the critical *A*_*starch*_ values and this change in the particle packing ability.

The area expected for a hexagonal packed layer of particles with a mean starch size (A_hex_) was calculated using equation 4 and is shown in Figure 2 by the dashed line. If the area where the tight packing region commenced in the surface pressure-*A*_*starch*_ isotherms of the native rice starch and the OSA modified rice particles are to the left of this line, then some of the particles may have been lost from the air/water interface to the subphase. The *A*_*starch*_ corresponding to the commencement of the tight packing region for the films of NRS, 0.6RS and 1.2RS particles were less than the A_hex_ value. The 1.8RS, 2.4RS, and 3.0RS particles gave films, where the *A*_*starch*_ corresponding to the commencement of the tight packing region was larger than A_hex_. Thus, the NRS, 0.6RS and 1.2RS particles are thought to have been less stable at the air/water interface than the 1.8RS, 2.4RS, and 3.0RS particles.

**Figure 3:**
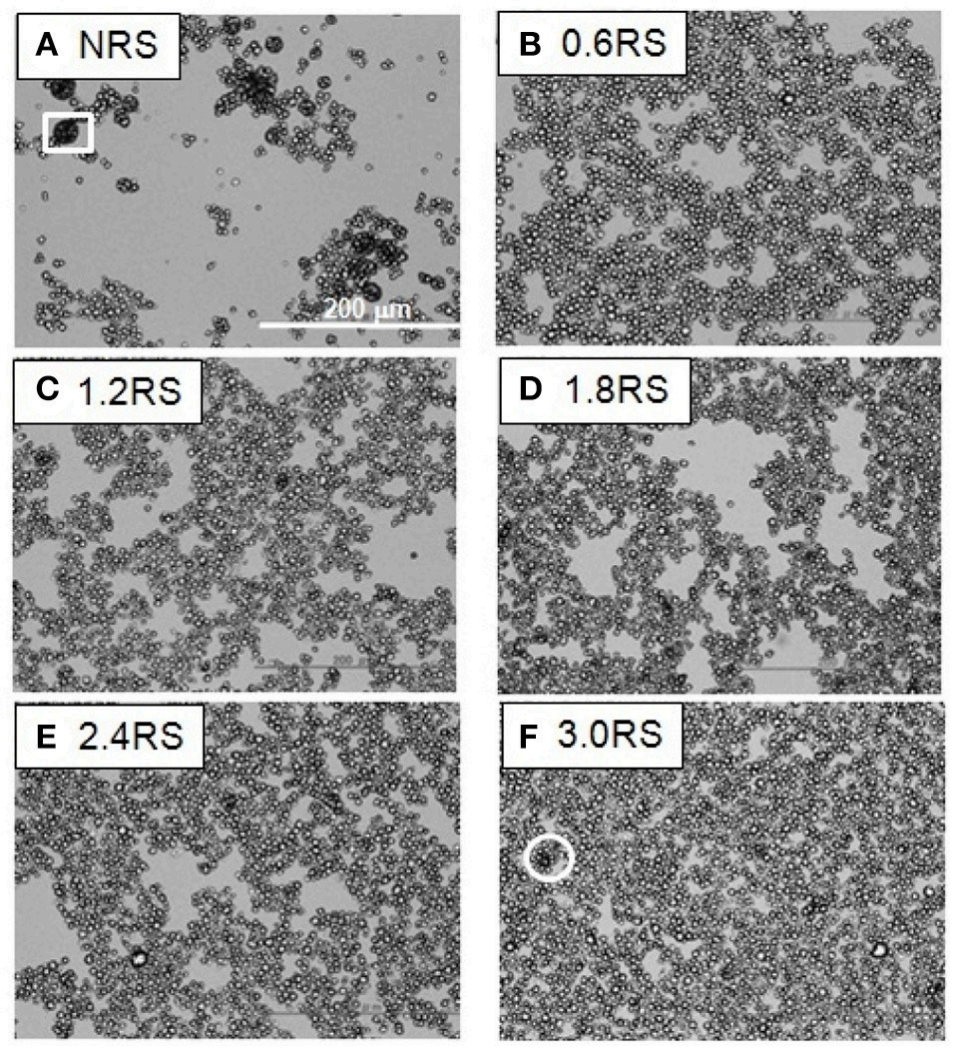
Optical microscope images of films of native and OSA modified starch particles compressed to ∏ = 5 mN/m at air/water interfaces. The white square shows an example of the large aggregates formed by the NRS particles. The white circles show examples of dark patches of closely packed OSA modified particles or aggregates in the films.

**Figure 4:**
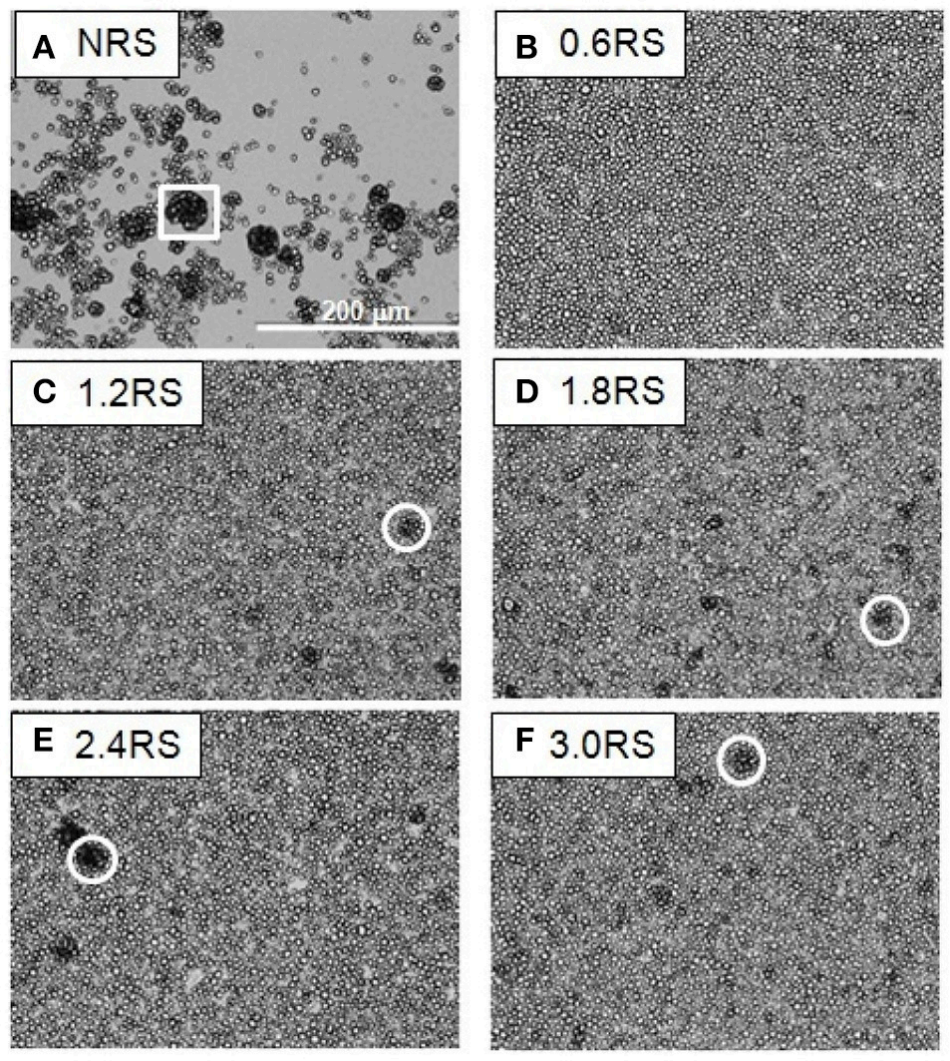
Optical microscope images of films of native and OSA modified starch particles compressed to ∏ = 15 mN/m at air/water interfaces. The white square shows an example of the large aggregates formed by the NRS particles. The white circles show examples of dark patches of closely packed OSA modified particles or aggregates in the films.

**Figure 5:**
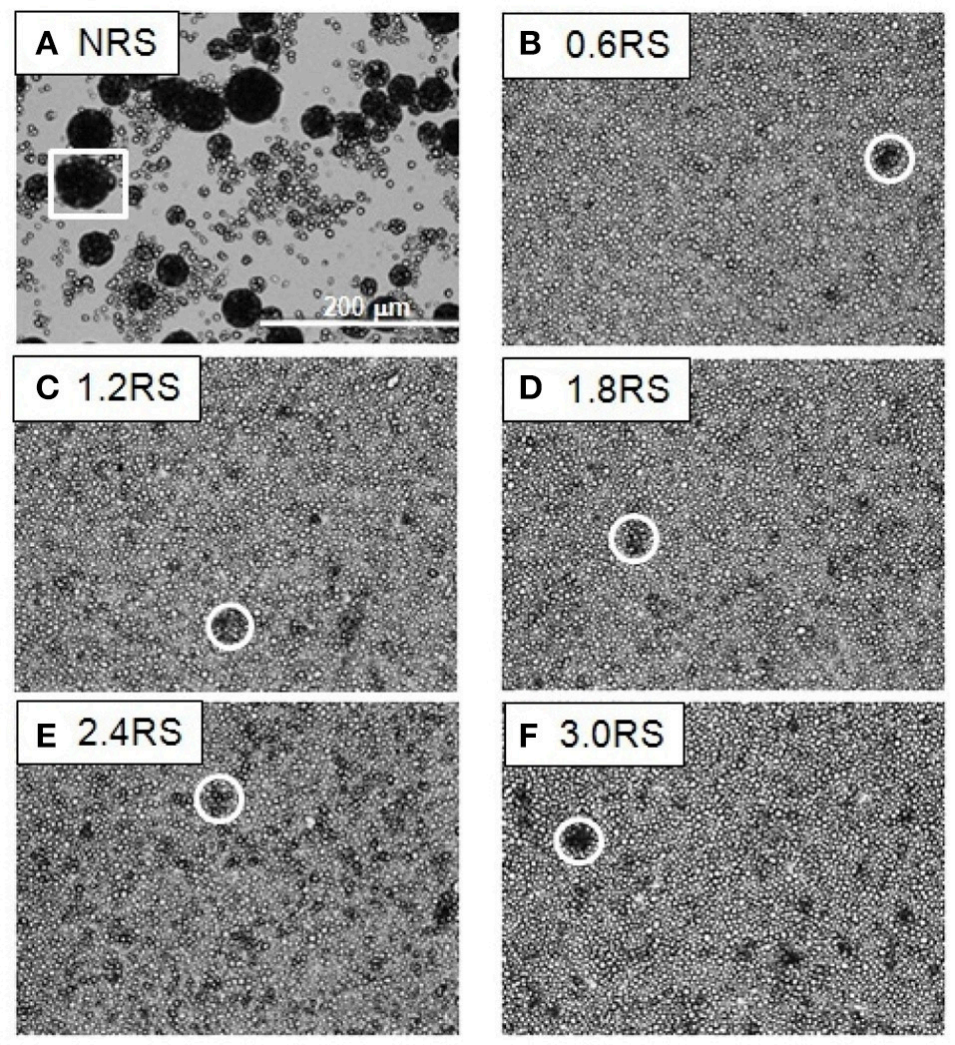
Optical microscope images of films of native and OSA modified starch particles compressed to ∏ = 25 mN/m at air/water interfaces. The white square shows an example of the large aggregates formed by the NRS particles. The white circles show examples of dark patches of closely packed OSA modified particles or aggregates in the films.

The effect of the structural modification of the starch particles on the packing of the starch particle at the air/water interface can be determined by using an optical microscope to image the starch particles in the Langmuir films at the air/water interface. Figures 3–**5** show the images of the Langmuir films of the native starch particles (NRS), and the OSA modified starch particles (0.6RS, 1.2RS, 1.8RS, 2.4RS, and 3.0RS) at air/water interfaces, when the films were compressed to surface pressure values of 5, 15, and 25 mN/m, respectively. These values were chosen as the surface pressure-*A*_*starch*_ isotherms indicated that these three values should allow the different packing densities to be studied. The NRS particles can be seen to aggregate to give large, 3-dimensional types of aggregates at the air/water interface, regardless of the surface pressure (see the white boxes in Figures 3–**5**). These aggregates were compressed as the film was compressed, resulting in a surface pressure increase with an *A*_*starch*_ decrease. A closely packed film of particles without holes could therefore not be formed at the air/water interface with the NRS particles due to this aggregation. This inability to form a closely packed film of particles may also be related to a loss of material to the subphase, due to the hydrophilicity of the native starch particles. In the case of the modified starch particles, films with holes were seen at Π = 5 mN/m. An increase in the degree of modification of the starch particles improved the packing of the particles in the film and consequently decreased the size of these holes seen in the film. The dark colored patches (see white circle in Figure 3F) in the film of 3.0RS particles showed that the 3.0RS particles could closely pack in the film or that multilayer aggregates were being formed in the film. The area occupied by these closely packed particles was much smaller than the ones seen in film of NRS particles. Thus, the mechanism causing these particles to closely pack or aggregate is thought to be different than that which caused the NRS particles to form the large aggregates in the interfacial film. Increasing the surface pressure to 15 mN/m reduced the size of the holes in the films of OSA modified starch particles (Figure 4). This result was explained by the fact that the air/water interfacial area available for the starch particles decreased as the surface pressure increased. This would cause the starch particles to pack closer together and to therefore reduce the area of bare air/water interfacial area. The number and size of the holes in the films decreased as the degree of modification was increased. The 1.2RS, 1.8RS, 2.4RS, and 3.0RS particles formed films without detectable holes. Areas of closely packed particles or aggregates were also observed at the air/water interface, see the dark patches of closely packed particles highlighted by the circles in the images in Figure 4. An increase in the surface pressure to 25 mN/m resulted in the 0.6RS, 1.2RS, 18RS, 2.4RS, and 3.0RS particles forming films of particles without detectable holes (Figure 5) and with dark patches of closely packed particles (see the white circles in Figure 5). The number of these dark patches appeared to increase with the degree of modification of the rice starch particles. Comparison of the number of the dark patches in the films of 3.0RS particles measured at different surface pressures showed that a surface pressure increase caused the number of these patches to increase.

The packing type assigned to each film of particles at the air/water interface by analyzing the surface pressure-*A*_*starch*_ isotherms was the same as those obtained by interpreting the optical images. This result indicates the reliability of the interpretation of the isotherms.

**Figure 6:**
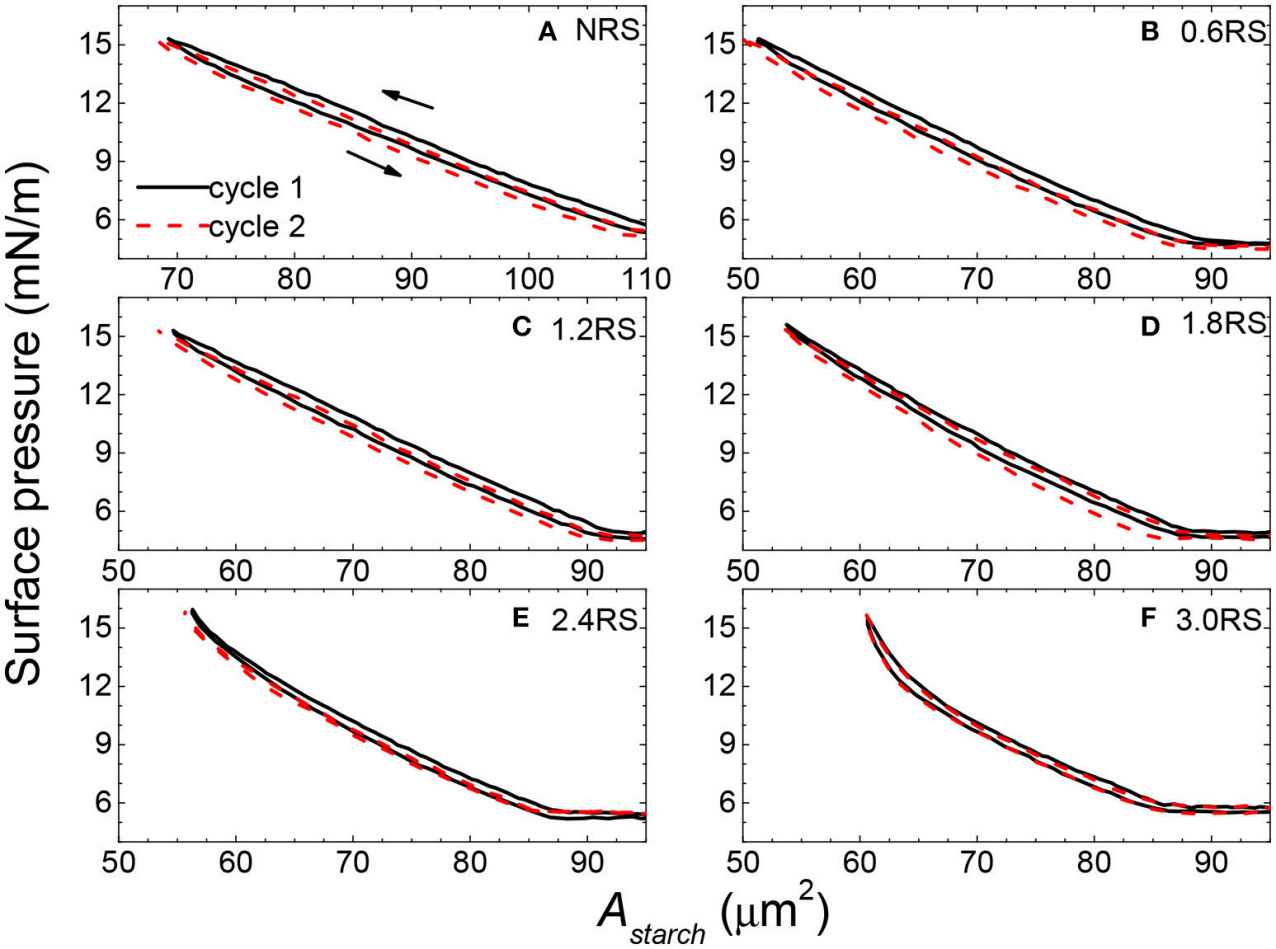
Two compression-expansion cycle surface pressure-area per rice starch particle (*A*_*starch*_) isotherm of films of native and OSA modified rice starch particles spread at an air/water interface. The black solid line and the red dashed line show the first and second cycle compression-expansion isotherms, respectively.

Information about the stability of the films of rice starch particles at the air/water interface and the interactions between the particles in the films can be obtained by measuring the compression-expansion cycle surface pressure-*A*_*starch*_ isotherms of the films. Figure 6 shows two cycle compression-expansion surface pressure-*A*_*starch*_ isotherms for the native and OSA modified rice starch particles. In the first cycle of all of the isotherms, the expansion isotherms shifted to lower surface areas of the compression isotherms, when the *A*_*starch*_ values measured at the same surface pressure were compared. This result indicates that the particles remained adhered to each other to some degree and did not immediately re-disperse at the air/water interface after compression, and/or that some of the starch particles were lost from the air/water interface to the subphase. Comparison of the isotherms measured in the first cycle with those measured in the second cycle also allows information about the strength of the adhesive force between the starch particles within the film and the stability of the particles at the interface to be obtained. If the isotherms move to lower surface areas, then the particles remained adhered to each other after the first compression, due to inter-particle attractions. Alternatively, some of the particles may have detached from the air/water interface into the water subphase. The compression-expansion isotherms shifted less to the left as the degree of modification of the rice starch particles was increased. Thus, the inter-particle attractions are thought to decrease with an increase in the degree of modification of the rice starch particles. An increase in the OSA modification is also thought to improve the stability of the particles at the air/water interface.

Increasing the degree of OSA modification of the rice starch particles increases the number of octenyl succcinate derivatives per glucose unit on the starch particle (Sweedman et al., 2013). The OSA modification is also reported to increase the hydrophobicity of the starch while also rendering the starch anionic (Nilsson and Bergenståhl, 2007a). This is because the octenyl succcinate substituent is hydrophobic and contains a carboxylic acid, which can also be negatively charged (Nilsson and Bergenståhl, 2007a), see Figure 1B.

In order to determine if the charge of the starch particles increased with an increase in the degree of the OSA modification, the zeta potential values of dispersions of native starch particles and dispersions of OSA modified starch particles in water were measured. These values are shown in Table 1. The zeta potential values increased from −19.8 mV for the NRS particles to −36.2 and −35.1 mV for the 2.4RS and the 3.0RS particles, respectively. In general, the zeta potential values became larger as the degree of the OSA modification was increased. The particles therefore tend to become more negatively charged as the degree of OSA modification increases. This increase is explained by the increase in the number of octenyl succcinate derivatives per glucose unit on the starch particles as the degree of the OSA modification is increased, where the negative charge is resulting from by the carboxylic acid substituent. The zeta potential of O/W emulsions stabilized by starch has also been reported to become more negative as the degree of OSA substitution is increased (Miao et al., 2014). The presence of the charge on the starch particle from the OSA modification would increase the inter-particle electrostatic repulsions in the film, causing the aggregation of the starch particles at the air/water interface to be reduced. The hydrophobicity of the OSA substituent gives the starch particle a degree of hydrophobicity, resulting in the starch particle showing surface active properties. It may also increase the inter-particle hydrophobic attractions within the film of particles at the air/water interface.

**Table 1:** Zeta potential (ζ) values measured of 100 ppm concentration of native or modified starches dispersed in water or aqueous solutions containing NaCl, where the concentration of NaCl is given by [NaCl].

**Sample**	**[NaCl] (mM)**	**ζ (mV)**
NRS	0	−19.8 ± 1.2
0.6RS	0	−27.8 ± 1.0
1.2RS	0	−34.1 ± 0.7
1.8RS	0	−32.5 ± 2.5
2.4RS	0	−36.2 ± 1.1
3.0RS	0	−35.1 ± 1.8
3.0RS	1	−29.7 ± 0.7
3.0RS	10	−24.9 ± 1.1
3.0RS	100	−8.4 ± 0.7

Attractive hydrophobic interactions can result between surfaces containing hydrophobic groups, such as long-chain alkanes, when they come in close proximity in aqueous systems. The strength of this hydrophobic interaction is thought to increase with the hydrophobicity of the surface. Increasing the degree of OSA modification of the starch particle would increase the number of the octenyl succcinate derivatives on the starch, which should cause the hydrophobicity of the starch particles to increase. Thus, the inter-particle hydrophobic attractions between the starch particles at the air/water interface were expected to increase with the degree of OSA modification. This therefore should have increased the aggregation of particles in the film at the air/water interface. A very strong attraction between the particles is expected to lead to a fractal type of aggregation (Joanicot et al., 1990; Mastushita et al., 1997), which therefore would increase the number of holes seen in the particle film at the air/water interface. Decreasing the surface area by closing the barriers causes the packing density of the particles in the film to increase. Thus, we would expect an increased attraction between the particles and aggregation with an increased compression of the film, due to capillary forces. The capillary forces would cause particles to aggregate more as the particles become more hydrophobic. The particles were therefore expected to aggregate more with an increased OSA modification. This was, however, not observed. The isotherms and optical images of the films of rice starch particles at the air/water interface shown in Figures 1–5 indicated that the packing of the particles in the film improved with the degree of modification of the rice starch particles and the surface pressure of the film. These results suggest that the presence of charged groups from the octenyl succcinate derivatives modulates the inter-particle hydrophobic interactions between the octenyl succcinate derivatives on the starch particles. This effect caused us not to observe this fractal type of aggregation.

**Figure 7:**
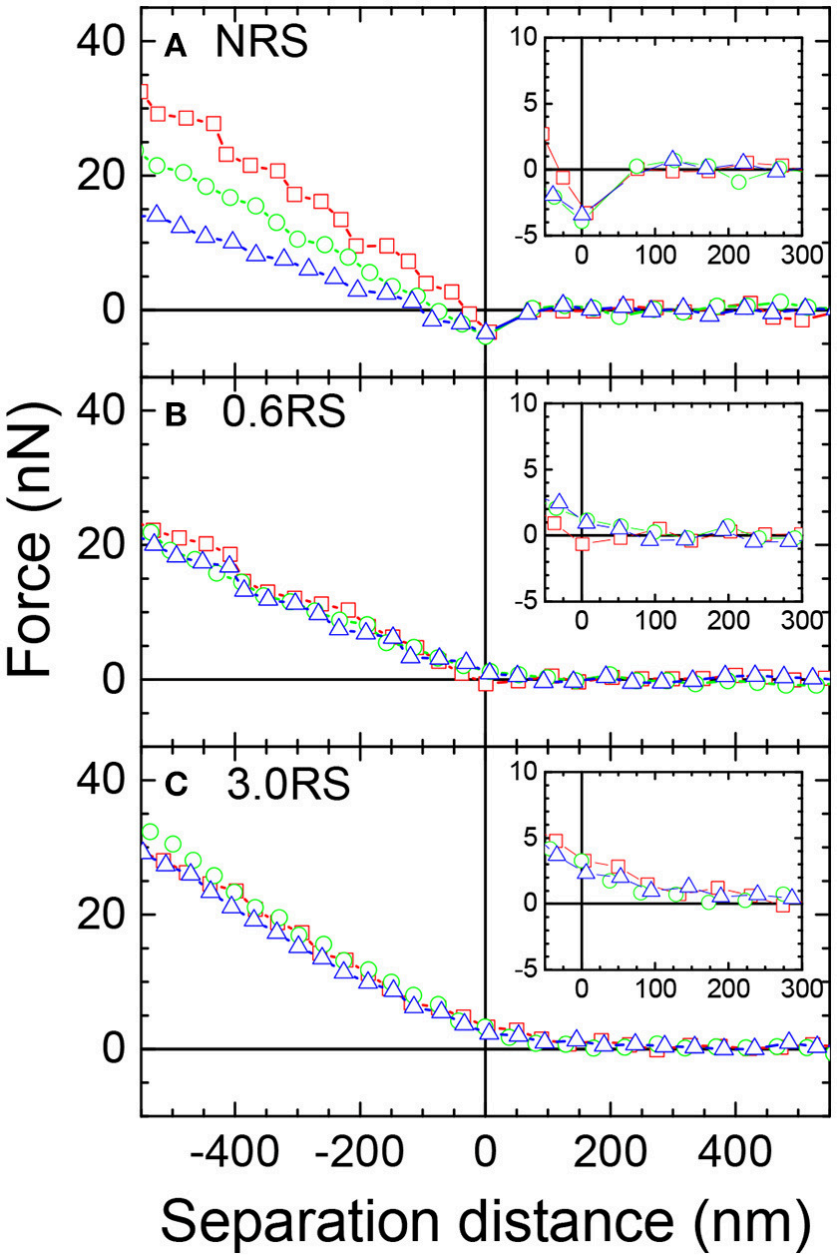
Example approach force curves between a starch particle (probe) in the water phase and a bare air/water interface or a starch film at an air/water interface, where the same particle type used to prepare the films was used as the probe. Red open squares: starch particle film compressed to Π = 5 mN/m; green open circles: starch particle film compressed to Π = 10 mN/m; blue open triangles: starch particle film compressed to Π = 15 mN/m. The insets show the force vs. separation distance curves on a magnified scale, showing the shift from the attraction to repulsion more clearly.

The difference in the physical properties of the films of the native and modified starch particles at the air/water interface was further investigated by measuring the force-distance curves between a starch particle attached to an AFM cantilever (probe) in the water phase and a film of starch particles at the air/water interface. The same starch particle type that was used to form the film of particles was used as the probe. Forces were measured for the NRS, 0.6RS, and 3.0RS systems, in order to determine the effect of modifying the rice starch structure and the degree of modification. Figures 7A–C show example approach force curves for the NRS starch particles, the 0.6RS starch particles, and the 3.0RS starch particles, respectively, when the films were compressed to ∏ = 5, 10, and 15 mN/m. The zero of the separation distance in the approach force curves was set as the distance where the linear contact region commenced, i.e., the distance where the probe started to be in contact with the particle films at the air/water interface. An attraction was observed in the approach force curves for the NRS particles for all the surface pressures, while the approach force curves for 0.6RS and 3.0RS gave a repulsion. The insets in Figure 7 show the force vs. separation distance curves on a magnified scale, showing the shift from the attraction to repulsion more clearly. The strength of this repulsion increased with the degree of modification of the rice starch particle. Increasing the degree of modification of the rice starch particles increases the number of octenyl succcinate derivatives per glucose unit on the starch particle. The increase in repulsion with the degree of modification of the rice starch particles can therefore be explained by this increase in number of octenyl succcinate derivatives, which are charged. The slope of the approach force in the linear contact region in the approach force curves changed with the degree of modification and surface pressure. The stiffness (*S*_*N*_) of the film of particles at the air/water interface was calculated from the slope of this region. An increase in the slope of this region indicates an increase in the interface stiffness. The retract force curves (not shown) showed an adhesion, the strength of this adhesion was calculated to give the adhesive force (*F*_*ad*_).

**Figure 8:**
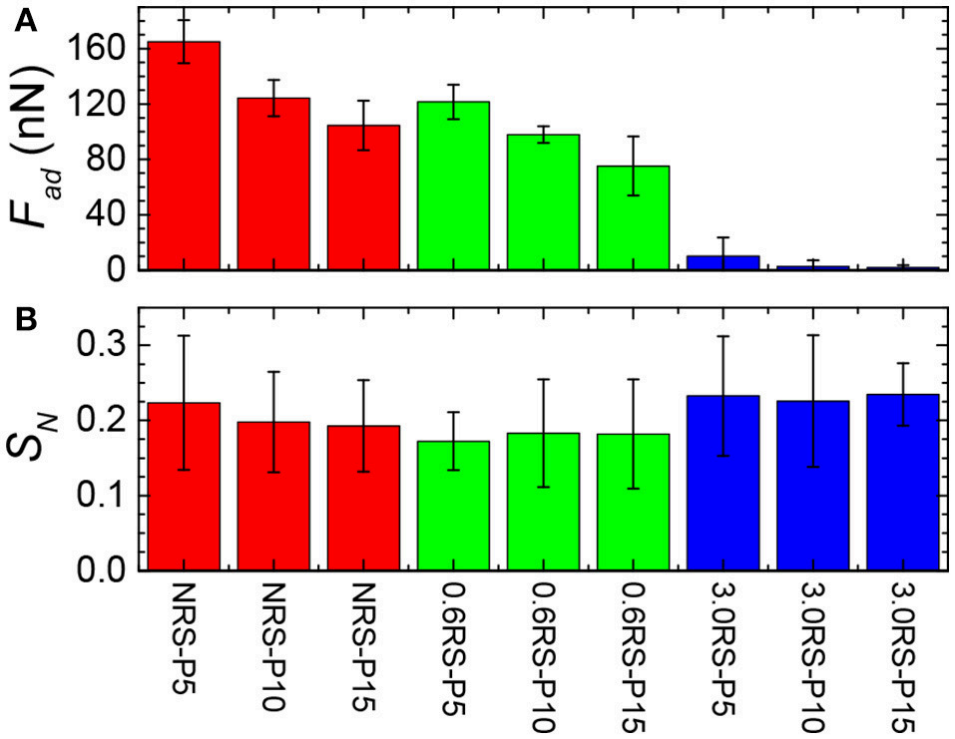
Effect of degree of modification of starch particle on the adhesion (*F*_*ad*_) of the starch particle to the film composed of the same starch particles at an air/water interface and the stiffness of the film (*S*_*N*_). **(A)** Adhesive force; **(B)** stiffness of the films at the air/water interface. NRS-P5: Film of NRS particles compressed to Π = 5 mN/m; NRS-P10: Film of NRS particles compressed to Π = 10 mN/m; NRS-P15: Film of NRS particles compressed to Π = 15 mN/m; 0.6RS-P10: Film of 0.6RS particles compressed to Π = 5 mN/m; 0.6RS-P10: Film of 0.6RS particles compressed to Π = 10 mN/m; 0.6RS-P15: Film of 0.6RS particles compressed to Π = 15 mN/m; 3.0RS-P10: Film of 3.0RS particles compressed to Π = 5 mN/m; 3.0RS-P10: Film of 3.0RS particles compressed to Π = 10 mN/m; 3.0RS-P15: Film of 3.0RS particles compressed to Π = 15 mN/m.

Figure 8A shows the effect of the degree of modification of the rice starch particles and the surface pressure of the film of particles at the air/water interface on the adhesive force (*F*_*ad*_) measured between a rice starch particle in the water phase and the film of particles at the air/water interface. The adhesive force tended to decrease with an increase in the degree of modification of the rice starch particles and with an increase in the surface pressure of the film of particles at the air/water interface. A decrease in the adhesive force indicates that the attractive forces between two starch particles separated by water have decreased. The introduction of an inter-particle electrostatic repulsion could explain this decrease in the adhesive force. The increase in number of charged octenyl succcinate derivatives on the rice starch particles with an increased degree of modification of the rice starch particles could cause this increase in the electrostatic repulsion between two rice starch particles. Additionally, compressing the film, i.e., decreasing the separation distance between the particles in the film, would increase the charge density of the film, causing the magnitude of the electrostatic repulsion to increase. The surface pressure was observed to increase with the film compression. Thus, the adhesive force may also have decreased with a surface pressure increase, due to this increased electrostatic repulsion.

Figure 8B shows the effect of the degree of modification of the rice starch particles and the surface pressure of the film of particles at the air/water interface on the stiffness (*S*_*N*_) of the film of particles at the air/water interface. There was only a small change in the measured surface stiffness between systems formed using the NRS, 0.6RS, and 3.0RS particles, when the same surface pressure values were compared. The stiffness of the films of 0.6RS particles was seen to be a little less than that of the film formed by the NRS particles. The stiffness of the film of 3.0RS particles was, however, a little greater than that of the films of NRS or 0.6RS particles. The lack of change in the stiffness for the particle types is thought to be related to the surface pressures used in the MPIA experiments. If the interface was held at a high enough surface pressure, then there would have been little flexibility in the interface. The forces measured would then have been dominated by the probe particle and the immediate particle in the interface that it contacts. This would have caused the influence of the surrounding network to be reduced.

Figure 8B also showed that the stiffness of the films of particles tended to decrease with a surface pressure increase for the NRS particles. The stiffness, however, either increased with the surface pressure or was independent of the surface pressure for the 0.6 RS and 3.0 RS particles. This change in the stiffness with surface pressure tendency indicates that the mechanism controlling the stiffness of the air/water interface in the presence of a film of particles were different for the native and modified starch particles.

The stiffness of a film of particles at the air/water interface is controlled by the surface tension of the interface, the stiffness of the particles, and continuity or integrity of the film. The stiffness of an interface decreases with the surface tension (or increased surface pressure) of the film. A film of loosely packed particles would contain areas of air/water interface that are bare and areas that are covered by particles. The size of the area of the air/water interface that is bare and therefore not covered by particles decreases with an increase in the particle packing density. As the surface tension of a bare air/water interface is higher than the surface tension of an air/water interface with adsorbed matter, the surface tension of a film of particles at the air/water interface would decrease with a surface pressure increase. This would cause the stiffness of the air/water interface with the particles to decrease with a surface pressure increase. The stiffness of an interface would also increase with an increased stiffness or hardness of the material adsorbed at the air/water interface. A change in the stiffness of the particles with the modification of the starch particles would therefore also affect the stiffness of the film of particles at the air/water interface. Starch gels made from OSA starch are reported to be softer than starch gels made from native starch (Ovando-Martinez et al., 2017). Thus, the stiffness of the starch particles is expected to decrease with an increase in the degree of OSA modification. The stiffness of the interface is also affected by the discontinuity of a film of particles at an air/water interface. The particles in a film of particles without holes would be capable of being moved more by a particle colliding with it from the bulk water than a film of particles without holes, causing its stiffness to be less than a film without holes (Azakami et al., 2017).

The decrease in the stiffness of the film of NRS particles at an air/water interface with a surface pressure increase shows that the surface tension effect affected the stiffness of that film. This result is also supported by that fact that the optical images of the films of NRS particles at air/water interfaces showed areas of bare water surface and areas covered by large aggregates of particles. A film of closely packed particles was not formed by the NRS particles, indicating that the film also contained a large degree of discontinuity, which would also tend to decrease the stiffness of the film.

The stiffness of the films of 0.6RS and 3.0RS particles did not decrease with the surface pressure. This result indicates that the surface tension effect was not the determining factor controlling the film stiffness for these particle types. The 3.0RS film was also seen to give a stiffer film than the 0.6RS film. As the stiffness of the starch particles are expected to decrease with an increase in the degree of OSA modification, this increase cannot be explained by the change in the stiffness of the actual starch particle resulting from a change in the degree of OSA modification.

An increase the surface pressure of a film of OSA modified rice starch particles at the air/water interface caused the particles to pack closer in the film and the number of holes in the film to decrease. An increase in the degree of OSA modification also decreased the number of holes in the film and allowed the particles to pack closer in the film. The increase in the stiffness of the film with the degree of OSA modification and surface pressure increase is therefore explained by the decrease in the number of holes in the film of particles at the air/water interface, due to an improved ability of the particles to pack in the film. This improved packing is explained by the increase in the inter-particle electrostatic repulsions resulting from the OSA modification. The particles in a film of loosely packed particles would be moved by the starch particle probe in the bulk water that is brought in contact with the film of starch particles. This lateral movement of particles within the film would cause the film to be disturbed and partially broken. The particles in a film that is made up of closely packed particles would be less capable of moving laterally at the air/water interface, due to a lack of free area and the presence of the inter-particle electrostatic repulsions acting within the film. Thus, the film would be less perturbed by the starch particle probe in the bulk water, causing this film to be stronger than the film with aggregating particles and holes. Thus, the stiffness increase with a surface pressure increase or an increase in the degree of OSA modification is explained by a decrease in the discontinuity in the film of particles at the air/water interface, i.e., a decrease in the number of holes in the film of particles. The increased surface pressure caused the particles to be pushed together, resulting in a more homogenous film without holes.

### Effect of the addition of salt to the physical properties of films of 3.0RS particles at an air/aqueous interfaces

The results of the above section suggested that the well-dispersed and tight packing film of 3.0RS particles was obtained due to the presence of charged groups on the starch particle, which were introduced due to the OSA modification of the starch particles. If the starch particles are forming the tight packing film without holes due to the inter-particle electrostatic repulsions resulting from these charged groups, then the introduction of salt into the water subphase should change these inter-particle interactions.

**Figure 9:**
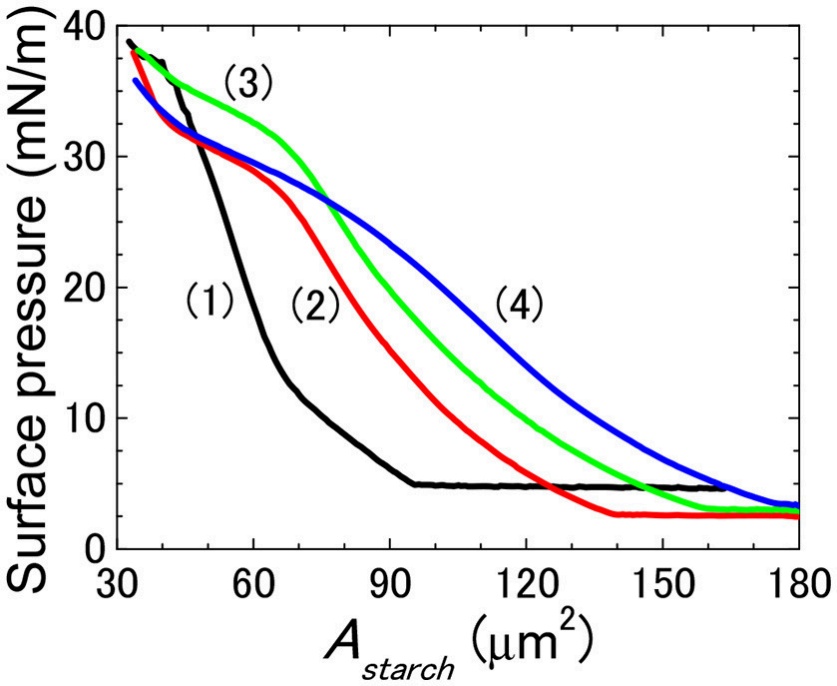
Effect of adding NaCl to the water to the surface pressure-*A*_*starch*_ isotherms of films of 3.0RS starch particles at air/aqueous interface. Water subphase: black solid line (1); 1 mM NaCl subphase: red solid line (2); 10 mM NaCl subphase: green solid line (3), and 100 mM NaCl subphase: blue solid line (4).

The effect of adding salt to water on which the particles formed the films was firstly investigated by measuring the surface pressure-*A*_*starch*_ isotherms for the films of 3.0RS particles spread on water, and on aqueous solutions containing 1, 10, and 100 mM NaCl (Figure 9). The isotherms are seen to shift to larger *A*_*starch*_ values for surface pressures < ~25 mN/m, as the concentration of salt in the water increased. The isotherms converged to the same area per 3.0RS particle value for high surface pressure values (>35 mN/m), i.e., highly compressed films.

The surface tension of air/aqueous solutions containing NaCl can be calculated to increase by 0.28% when the concentration of NaCl was increased from 0.1 to 100 mM, if the values of the apparent relative surface tensions of air/NaCl aqueous solutions are compared (Jones and Ray, 1941). As the surface tension change accompanying an NaCl concentration increase is small, the shift in the isotherms due to the NaCl concentration increase is thought to be due to another reason.

**Figure 10:**
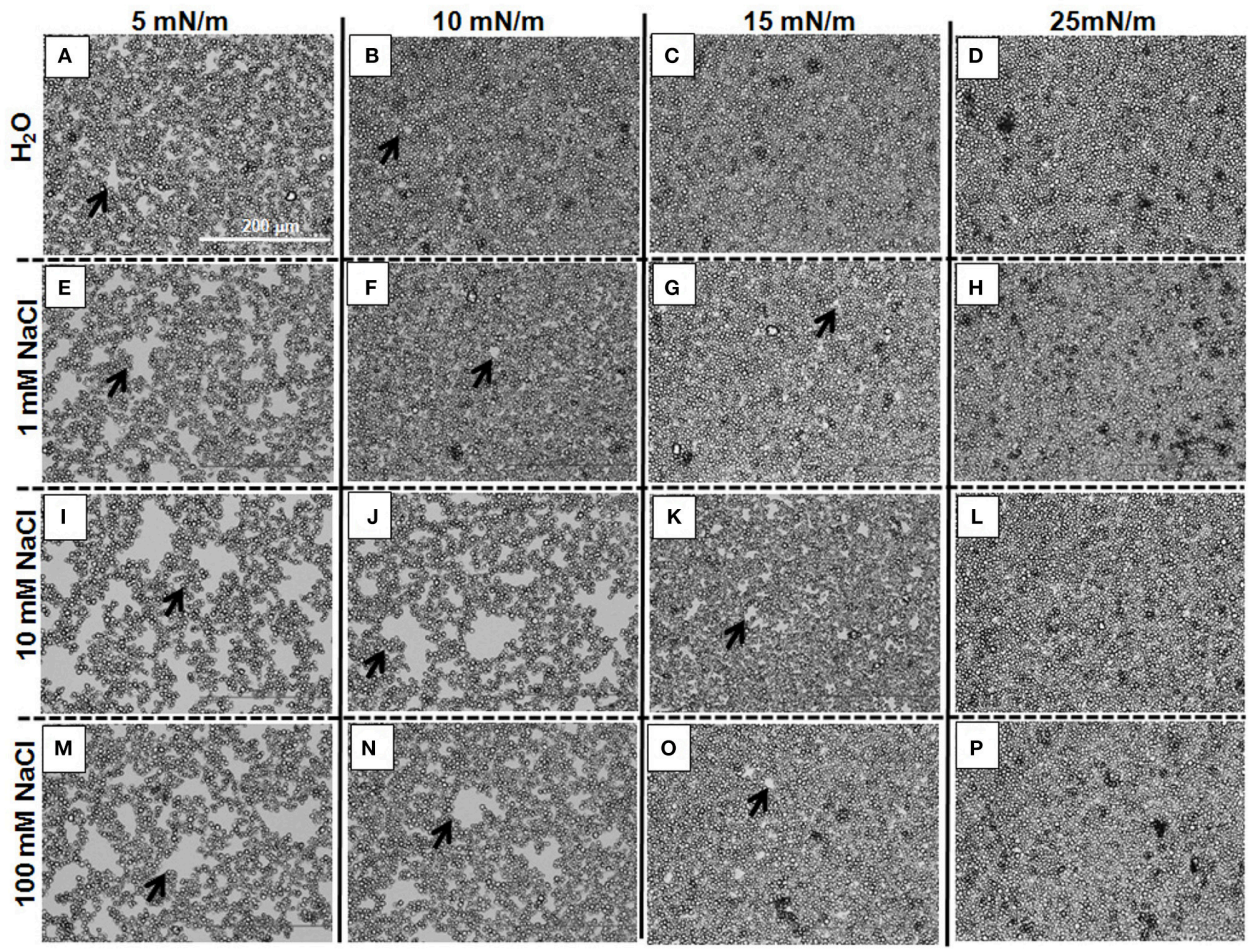
Optical microscope images showing the effect of adding NaCl to the water of films of 3RS particles at air/aqueous interfaces compressed to 5, 10, 15, and 25 mN/m. Water subphase: **(A–D)**; 1 mM NaCl subphase: (**E–H)**; 10 mM NaCl subphase: **(I–L)**; 100 mM NaCl subphase: **(M–P)**. Π = 5 mN/m: **(A,E,I,M)**; Π = 10 mN/m: **(B,F,J,N)**; Π = 15 mN/m: **(C,G,K,O)**; Π = 25 mN/m: **(D,H,L,P)**. The black arrows show examples of holes in the films of particles at the air/aqueous interfaces.

Information as to why the isotherms shift to larger *A*_*starch*_ values with a salt concentration increase was obtained by using an optical microscope to observe the films of 3.0RS particles at the different subphases. Figure 10 shows the images obtained for the films of 3.0RS particles on subphases of water, 1, 10, and 100 mM NaCl, when the films were compressed to 5, 10, 15, and 25 mN/m. The size and number of the holes in the films (examples shown by the black arrows in Figure 10) tended to increase with the concentration of salt in the water subphase. The size and number of these holes also decreased with a surface pressure increase. Few holes were visible at the high surface pressure value of 25 mN/m. These holes are thought to be formed due to the aggregation of particles within the films at the air/aqueous interface. An increase in the number and size of holes can be explained by an increase in the particle aggregation. The holes were no longer visible when the films were highly compressed, i.e., at high surface pressures. The shift in the isotherms to higher *A*_*starch*_ values with a salt concentration increase can be explained by the presence of these holes.

**Figure 11:**
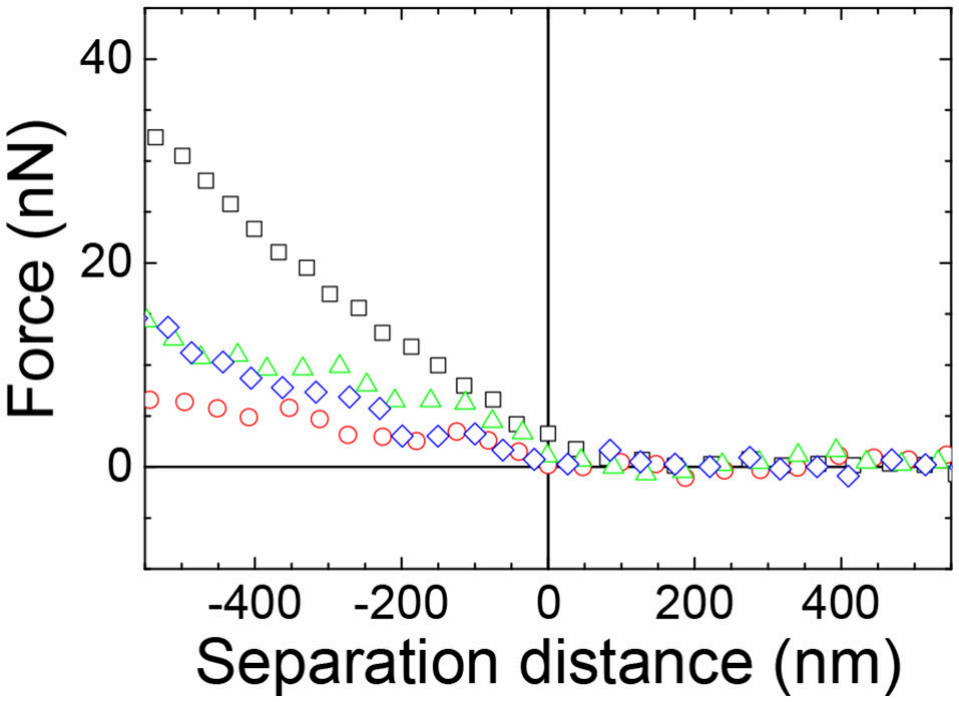
Example approach force curves between a 3.0RS starch particle (probe) in the aqueous phase and a 3.0RS starch film at an air/aqueous interface compressed to 10 mN/m showing the effect of adding NaCl to the water. Water subphase: black squares; 1 mM NaCl subphase: red circles; 10 mM NaCl subphase: green triangles; 100 mM NaCl subphase: blue diamonds.

The effect of the presence of the holes formed at higher ionic strength of the aqueous subphase on the stiffness of the films of particles was next investigated. The force curves between a 3.0RS particle in the subphase and films of 3.0RS particle compressed to 10 mN/m at air/aqueous interfaces were recorded with 0, 1, 10, and 100 mM NaCl in the subphase (Figure 11). The surface pressure of 10 mN/m was chosen, as this surface pressure best showed the influence of the salt presence. A repulsive force was observed, regardless of the concentration of NaCl in the subphase. The slope of the approach force in the linear contact region in the approach force curves changed with the concentration of NaCl added to the water. The retract force curves (not shown) showed a repulsion, causing the strength of the adhesion to be very small or negligible.

**Figure 12:**
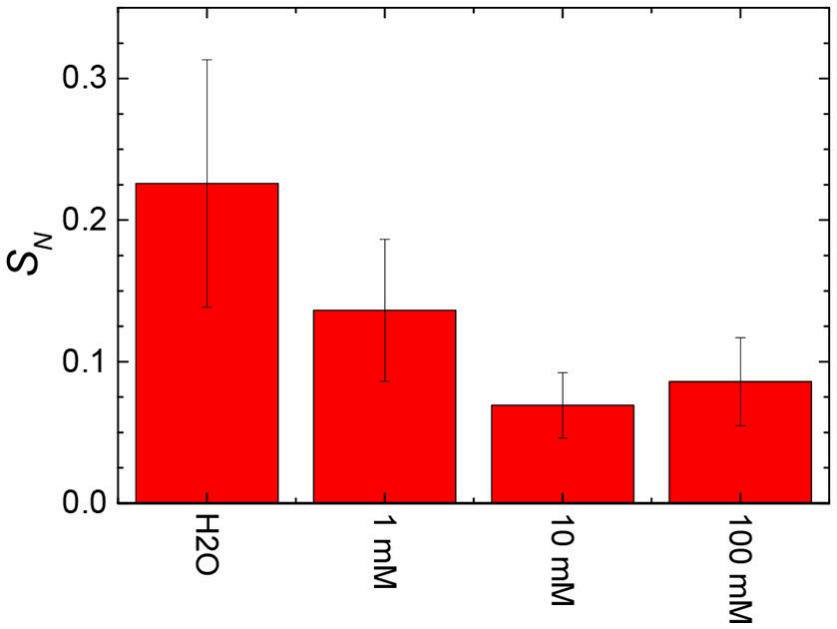
Effect of the addition of salt to the water on the stiffness of films (*S*_*N*_) of 3.0RS particles compressed to Π = 10 mN/m at air/aqueous interfaces.

The stiffness values obtained from the slope of the linear contact area of the approach force curves were calculated and are shown in Figure 12 as a function of the concentration of NaCl in the subphase. The stiffness values tended to decrease with an increase in the NaCl concentration from 0 to 10 mM. An increase in the concentration from 10 to 100 mM NaCl appeared to slightly increase the stiffness of the film of particles at the air/aqueous interface.

In general, the stiffness of the film of particles tended to decrease with an increase in the number and size of holes in the film of particles. The decreased stiffness can be explained by an increased discontinuity in the particle film, due to the presence of the holes.

Previous studies investigating the physical properties of a monolayer of charged surfactants at air/aqueous interfaces showed that the addition of salt to the water subphase changed the packing of the monolayer and caused the stiffness of the interface to increase with high NaCl concentrations (McNamee et al., 2012). This increase was explained by either the increased stiffness of the air/aqueous interface itself for highly concentrated salt solutions and/or an increased dehydration of the monolayer. Thus, the slight increase in the stiffness of the film of particles at the air/100 mM NaCl interface that was seen in this present study is thought to be related to the dehydration of the interface by the high salt concentration. Here we also note, though, that the starch particles themselves are expected to be hydrated.

The formation of holes due to the increased aggregation of particles within the films at the air/aqueous interface with a salt concentration increase can be explained by a decrease in the magnitude of the inter-particle electrostatic repulsions. The electrostatic repulsion between two charged surfaces separated by an aqueous solution decreases with an ionic strength increase due to an increase in the screening of the charged surfaces (Israelachvili, 2011). This decrease in the electrostatic repulsion can also be understood by the decrease in the Debye length (Israelachvili, 2011) of a charged surface in aqueous solutions containing NaCl, when the NaCl concentration was increased from 1 to 100 mM. The Debye length for an aqueous solution containing 1, 10, and 100 mM NaCl can be calculated to be 9.62, 3.04, and 0.96 nm, respectively. The effect of adding NaCl to the water on the charge of the 3.0RS particles was also determined by measuring the zeta potentials of the 3.0RS particles dispersed in water and in aqueous solutions containing NaCl. Table 1 shows that the zeta potential of the 3.0RS particles decreased from −35.1 to −8.4 mV, as the concentration of NaCl was increased from 0 to 100 mM. Thus, NaCl was concluded to screen the charges on the 3.0RS particles.

The force acting between two particles is the resultant of the attractive and repulsive forces. The effect of the inter-particle attractive forces would therefore be more prevalent in the total forces acting between the two particles in the presence of salt. Other studies have also shown that the addition of salt to starch granule stabilized emulsions for starch modified with OSA to 4.66% increased the size of the droplet, which was explained by an increased droplet aggregation (Rayner et al., 2012a). These results also therefore indicate that the addition of salt can increase the attractions in the system. Screening of the charge on the particles by an increased NaCl concentration would cause the rice particle to appear more hydrophobic. The lateral capillary force would cause particles to aggregate more as the particles become more hydrophobic.

Modification of the rice starch particles caused octenyl succcinate derivatives to be introduced on the starch particle, which contained both charge and hydrophobicity. In the absence of salt in the water subphase, the presence of the charge reduced the aggregation of the particles at the air/aqueous interface. This was explained by the inhibition of the inter-particle hydrophobic attractions resulting from the charge of the octenyl succcinate derivatives. The addition of salt to the water subphase caused these charges to be screened. As a result, the hydrophobic octenyl succcinate derivatives could have come into contact closer, causing the strength of the inter-particle hydrophobic attractions to increase. These attractions would have caused the particles to aggregate and form islands at the air/aqueous interface. Holes would be present in the film of particles, when the particles are not compressed enough to remove the spaces between the islands. The stiffness of the film of particles with holes is lower than one without holes. It would therefore be necessary to compress the films to higher amounts in order to achieve a dense packing density, if a stiff and strong film of particles is required with OSA modified starch particles used at air/aqueous interfaces containing salts.

## Conclusions

Native rice starch particles formed large aggregates at air/water interfaces. A two-dimensional film of particles could not be formed with these particles at an air/water interface. The modification of the rice starches particles decreased the aggregation of the starch particles, due to the introduction of a charged group on the starch particle. Increasing the degree of modification improved the particle packing within the film of particles at the air/water interface, due to the increase in the magnitude of the inter-particle electrostatic interactions arising from the greater number of charged groups on the particles. The stiffness of the film also increased with the degree of modification, due to this improved particle packing. Introduction of salt to the water phase caused the particles to aggregate and form holes within the film, due to screening of the charged groups on the starch particles. The presence of these holes decreased the stiffness of the films.

The packing and stiffness of films of starch particles formed at hydrophobic/hydrophilic interfaces in the absence of salt can be improved by increasing the degree of modification of the starch particles. In the presence of salt, the films of particles must be compressed to give a high packing density, in order to remove holes in the films of the particles. Such holes would decrease the stiffness of the films.

## Author contributions

CM: Designed the study, performed the research, wrote the manuscript; YS and IF: Performed the research; MK: Provided new methods; BW: Prepared the starch particles; TN, MR, and AM: Designed the study and wrote the manuscript; and all authors approved the revisions.

### Conflict of interest statement

The authors declare that the research was conducted in the absence of any commercial or financial relationships that could be construed as a potential conflict of interest.
